# Development of a Multiplexed LC-MS/MS Assay for the Quantitation of Podocyte Injury Biomarkers Nephrin, Podocalyxin, and Podocin in Human Urine

**DOI:** 10.1021/acs.jproteome.4c00751

**Published:** 2024-12-09

**Authors:** Carlos A Morales-Betanzos, Stephen P Berasi, Joel D Federspiel, Hendrik Neubert, Mireia Fernandez Ocana

**Affiliations:** † Pfizer Inc., Andover, Massachusetts 01810, United States; ‡ Pfizer Inc., Cambridge, Massachusetts 02139, United States

**Keywords:** kidney, CKD, glomeruli, podocytes, biomarkers, LC-MS, targeted MS

## Abstract

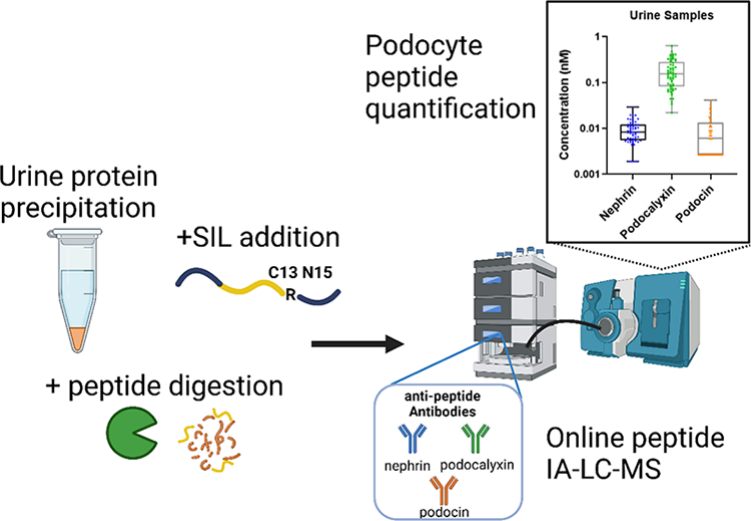

CKD is frequently diagnosed only after a significant progression. GFR is the most common indicator of kidney function but is limited in detecting early CKD cases and distinguishing glomerular, tubular, and global CKD. Aiming to provide a glomeruli specific biomarker assay, we developed a peptide immunoaffinity targeted mass spectrometry method for the quantitation of three podocyte specific proteins in human urine: nephrin, podocalyxin, and podocin. Proteins in urine were precipitated, stable isotope labeled peptide standards incorporated, and digested with trypsin. Target peptides were enriched using an online antibody column prior to LC-MS/MS. The performance metrics for nephrin, podocalyxin, and podocin were evaluated: The lower limits of quantitation were 3.8, 22.0, and 5.4 pM, respectively. The intraplate relative error (RE) was within ±10.6%, ± 10.4%, and ±16.1%, and coefficient of variation (CV) was ≤27.2%, ≤ 14.1%, and ≤20.7% accordingly. The interplate RE was within ±7.0%, ± 3.8%, and ±3.0%, and CV was ≤17.2%, ≤ 12.1%, and ≤20.0% for the three analytes. The urinary nephrin, podocalyxin, and podocin concentrations in 60 healthy volunteers and 20 disease samples was measured, thereby establishing the basal levels of these protein and enabling future evaluation of their roles as noninvasive biomarkers of glomerular injury in the clinic.

## Introduction

Chronic kidney disease (CKD) is defined as the gradual loss of kidney function over time and quantified as estimated glomerular filtration rate (eGFR) less than 60 mL/min per 1.73 m^2^, persisting for 3 months or more.^1^ CKD often results in renal failure, with the need for replacement therapy (dialysis or transplantation). CKD prevalence worldwide is estimated to be 8–16%, with patient numbers rising due to increasing overall age and hypertension and diabetes prevalence. CKD is most frequently diagnosed after abnormal findings in a routine urinalysis or blood test with many patients remaining asymptomatic until the disease has significantly progressed. GFR is the most established indicator of kidney function and can be measured using contrast agents or estimated as the eGFR. GFR measurements can be limited by low sensitivity in detecting early CKD and potential discrepancies when estimating using either creatinine (Cr) or cystatin C (CysC) measurements.^2^ Identifying biomarkers of kidney injury that could provide information on the state of glomerular injury, emphasizing the detection of early CKD with high specificity, would be of great value to patients and health care providers, as well as aid in clinical trial design and prosecution for new investigational therapies.

Along with GFR and eGFR, measurement of urinary protein (proteinuria), commonly normalized as urine protein to creatinine ratio (uPCR) has been used to diagnose CKD onset and severity. In these approaches, total urinary proteins are measured with increasing amounts, typically correlating with disturbances in renal filtration. However, total protein measurements are limited in detecting early CKD stages due to the lack of specificity to distinguish glomerular, tubular, or systemic kidney damage that can occur at different disease stages or etiologies. Histopathologic assessment of a biopsy provides the definitive evidence of a specific CKD diagnosis; however, due to their invasive nature, they have limited use in longitudinal studies of disease progression.^3^

In 2018, six noninvasive biomarkers were qualified by the FDA; kidney injury molecule-1 (Kim-1), neutrophil gelatinase-associated lipocalin (NGAL), clusterin, osteopontin, N-acetylglucosamine, and cystatin C, to aid in the detection of tubular injury of the kidney in phase 1 trials. Kim-1 and NGAL are also used in nonclinical studies to detect tubular kidney damage.^4^ Thus, far, only a few biomarkers of glomerular damage have been described in investigations focused on proteins that are unique to the morphology and function of the glomerulus. Podocytes are highly specialized epithelial cells that line the outer layer of the glomerular basement membrane, having numerous actin-rich foot processes that interdigitate with neighboring podocytes to create filtration slits. These gaps are approximately 40 nm wide and are connected by a semiporous protein impermeable layer called the slit-diaphragm which operates as the central barrier of the glomerular filtration system.^5,6^ Proteins like nephrin, podocin, Neph1, TRPC6, and FAT1 are essential for the integrity of the slit-diaphragm, supported by other structural proteins like podocalyxin and synaptopodin that are localized across the cell body of the podocyte. Glomerular disease is characterized by podocyte injury manifesting in effacement of the foot processes and ultimately podocyte detachment with increased proteinuria as a consequence.^7^ Therefore, noninvasive measurement of urinary podocyte proteins as potential glomerular damage biomarkers is attractive due to their specificity and potential early detection compared to uPCR.^8^ Ideally, a suitable assay would allow the measurement of multiple podocyte proteins in a wide range of concentrations to simultaneously detect relatively higher abundance structural proteins and lower abundance slit diaphragm proteins.

Liquid chromatography-tandem mass spectrometry (LC-MS/MS) is an analytical technique that joins the separation power of chromatography with the high specificity of mass spectrometry. LC-MS/MS can also be further combined with affinity-based techniques like protein or peptide immunoaffinity to further enhance sensitivity and specificity.^9−12^

Previously, LC-MS/MS was used to detect podocyte specific proteins in the urine of human patients. Michon et al. detected nephrin, podocalyxin, and podocin in the urine of renal transplant patients.^13^ Martineau et al. detected podocalyxin and podocin in preeclampsia and Fabry disease urine samples with a lower limit of quantitation (LLOQ) of 56.9 and 63.3 pmol/L, respectively.^14^ Biarc et al. detected podocalyxin in healthy volunteers (HV) with a LLOQ of 1 ng/mL, which equates to 17.1 pmol/L, a little over 3 times more sensitive than Martineau.^15^ In these publications, endogenous podocalyxin was detected well above the LLOQ while podocin was sparingly detected in concentrations close to the assay LLOQ. A mass spectrometry method for the quantitation of kidney proteins including nephrin in urine was developed by van Duijl et al., however, was unable to detect nephrin in urine from 10 HVs and 20 acute kidney injury patients.^16^

In this work, we present the development and characterization of an LC-MS/MS assay for the quantitation of podocyte proteins nephrin, podocalyxin, and podocin in human urine in a single multiplex assay. The method employs online peptide immunoaffinity in combination with stable isotope labeled (SIL) standard peptides and LC-MS/MS. The assay performance was characterized and qualified before application to a cohort of 60 samples from HV and 20 disease samples from Focal Segmental Glomerulosclerosis (FSGS) and Minimal Change Disease (MCD) patients with a wide range of proteinuria levels.

## Methods

### Peptide Targets

Seven proteotypic peptides that uniquely represent four podocyte proteins of interest: two for nephrin, podocalyxin, and podocin, and one for synaptopodin were monitored with an LC-MS/MS method (Table S1 and S2). The target and SIL peptide sequences are listed in Table 1. For each peptide, an unlabeled “light” and SIL peptide standard were acquired from New England Peptide or Thermo Scientific. SIL peptides contained ^13^C and ^15^N labeled arginine (R^) or lysine (K^) as their C-terminal residues unless otherwise noted. The tryptic peptide sequences for each standard are flanked by 3–5 additional amino acids that are removed upon trypsin digestion to account for potential differences in the digestion efficiency during the assay procedure. Peptide concentration (10 μM) and purity (≥95%) were confirmed by the vendor using amino acid analysis. Peptides were stored at −80 °C and tested routinely before each analysis.

**Table 1:** LC-MS Peptide Information: Target Protein Name, Peptide Sequence, Abbreviation, Extended Stable Isotope Label (SIL) Information, Target Transition, Transition Summing Use (TS),^20^ and m/z Values for Light and SIL Peptides

Target	Peptide type	Peptide sequence	Abbreviation	Extended SIL sequence (^) = −13C, −15N	Top Transition	Light (Q1/Q3)	SIL (Q1/Q3)
Nephrin	Quantitative	ELVLVTGPSDNQAK	ELV	H2N-STFSRELVLVTGPSDNQAK^FTCKA–OH	+2Y9 (TS)	735.894/917.432	739.901/925.446
Podocin	Quantitative	APAATVVDVDEVR	APA	H2N-PGEPRAPAATVVDVDEVR^GSGEE–OH	+2Y9	671.354/1031.537	676.358/1041.545
Podocalyxin	Quantitative	LGDQGPPEEAEDR	LGD	H2N-VSDMKLGDQGPPEEAEDR^FSMPL–OH	+2Y9	706.818/999.438	711.822/1009.446
Synaptopodin	Confirmatory	VTPNPDLLDLVQTADEK	VTP	H2N-FVEKPKVTPNPDLLDLVQTADEK^RRQRD–OH	+2Y9	934.486/1018.505	938.493/1026.519
Nephrin	Confirmatory	DGLLLGPDPR	DGL	H2N-VQWAKDGLLLGPDPR^IPGFP–OH	+2Y6	526.79/654.357	531.794/664.365
Podocalyxin	Confirmatory	DDLDEEEDTHL.	DDL	H2N-DNLTKDDLDEEEDTHL^–OH	+2Y8	665.768/987.39	669.276/994.407
Podocin	Confirmatory	TQGSLPFPSPSKPVEPLNPK	TQG	H2N-SPSNRTQGSLPFPSPSKPVEPLNPK^KKDSP–OH	+2Y8	707.384/893.509	710.056/901.523

### Antipeptide Antibody Generation and Column Preparation

**Figure 1:**
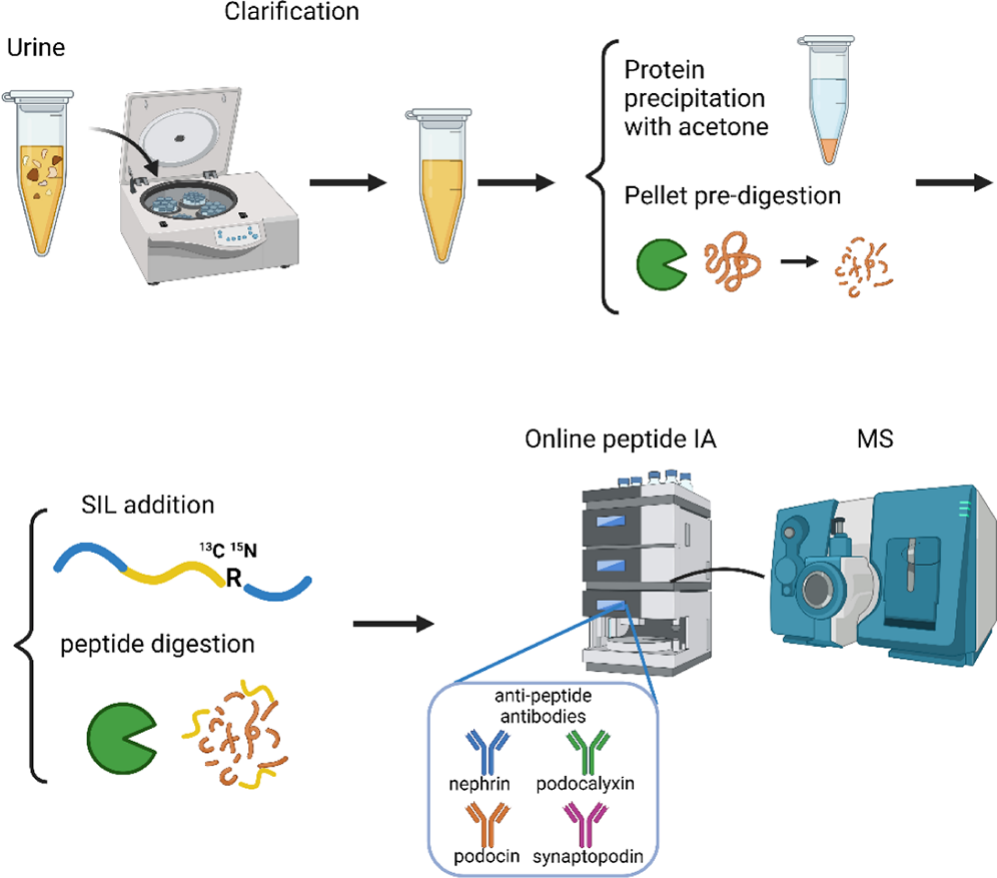
Urine preparation and Immunoaffinity LC-MS/MS workflow. Samples are clarified 1,000 × g for 5 min at 4 °C. 100 μL of clarified urine is precipitated with acetone and predigested overnight with trypsin. Extended stable isotope labeled (SIL) peptides are added to all standard, quality control solutions (QC) and samples before reduction, alkylation, and digestion. Peptides are analyzed with online peptide immunoaffinity, reverse phase chromatography, and tandem MS. Created in BioRender. Morales Betanzos, C. (2024) https://BioRender.com/x21t232.

Affinity purified rabbit polyclonal antibodies specific for nephrin (ELV and DGL), podocalyxin (LGD and DDL), podocin (APA and TQG), and synaptopodin (VTP) peptides were generated by Cambridge Research Biochemicals as previously described.^17,18^ Each antibody was generated in a single 25–60 mg lot, aliquoted and stored at −80 °C. Two antibody cartridges, one for podocalyxin and podocin, and one for nephrin and synaptopodin antibodies were generated as previously described^9−12,17,18^ and connected in series inside the LC system. Column preparation details and LC schematics are provided in the supplemental methods and Figure 1. The antibody recovery was tested before each analysis using peptide standards of known concentration as part of system suitability procedures.

### Calibration Standards (STD) and Quality Control (QC) Samples

Eleven calibration standards of decreasing concentrations (STD12–2) and one matrix blank (STD1) were prepared by diluting a pooled mixture of synthetic peptides or matrix blank in 1% rat serum in PBS as a surrogate matrix. The calibration range for nephrin was 7 to 0.0038 nM, for podocalyxin 40 to 0.0217 nM, and for podocin 10 to 0.0054 nM. Individual calibration standard concentrations are shown in Table S3. QC samples consisted of three pools of urine designated QC-A, -B, and -C with discrete concentrations of the target peptides (Table S4).

### Sample Preparation and LC-MS/MS

The general workflow consists of the following steps: urine clarification, protein precipitation, protein predigestion, SIL peptide addition, digestion, and online peptide immunoaffinity LC-MS/MS. The process is outlined in Figure 1 and detailed in the supplemental methods.

### Assay Performance Evaluation

To qualify the assay, three batch runs were independently conducted on separate days. Each plate contained a duplicate, combined calibration curve for all analytes consisting of 11 calibration standards of different concentrations, one matrix blank also in duplicate, and three QC samples (-A, -B, and -C) each in six replicates. All QC solutions and unknown samples underwent the same sample processing. At the end of the characterization, the nominal values for QC-A and -B were assigned as the average of 18 independent measurements. The value for QC-C was assigned as the nominal QC-B concentration plus a spike of light peptide of 0.066 nM for nephrin and 1 nM each for podocalyxin and podocin.

Intra- and interplate CV and RE were evaluated to establish the acceptance criteria for subsequent sample analyses. For assay characterization runs to pass, QCs should exhibit a CV less than or equal to 30%, and two-thirds of the QC samples at each level must show a RE less than or equal to ±30% of the nominal value established during the 3-day validation. Calibration standards for each analyte must show RE less than or equal to ±30% for at least one replicate per standard concentration and for at least 80% of the total replicates per curve to pass. The same criteria were used to evaluate sample stability at RT and 4 °C for up to 4 h, LC autosampler stability at 10 °C for up to 96 h, and for up to three freeze/thaw cycles (one cycle per day) at −80 °C. These criteria are consistent with a tier 2 assay classification in the targeted mass spectrometry community guidelines.^19^ Due to the lack of ideal commercial reagent calibrators required for a tier 1 assay, we were not able to achieve that level of quantitation, but we were able to demonstrate tier 2 accuracy and precision for nephrin and go beyond tier 2 requirements for podocalyxin and podocin.

### Urine Samples

Sixty urine samples from HVs were purchased from BioIVT (Westbury NY, USA). Each sample was collected without filters and frozen fresh. Urine samples were divided by sex and two age groups (18–40 years and 41–80 years), each of the four groups consisting of 15 subjects. As per vendor SOP, all samples used in this study were collected after informed consent was obtained, in accordance with Institutional Review Board (IRB) approval, and in adherence to the Declaration of Helsinki. All subjects were verbally screened for no history of kidney-disease-related medication (common medications to avoid included diabetes, preeclampsia, and hypertension drugs: insulin, ACE inhibitors, and angiotensin receptor blockers). Additionally, 18 clarified urine samples from patients with FSGS and two with MCD, and corresponding uPCR measurements were kindly provided by Dr. Jeffrey Kopp from the National Institute of Diabetes and Digestive and Kidney Diseases (NIDDK) and collected under applicable laws, regulations, and ordinances.

## Results

### Assay Performance

Seven podocyte peptides were targeted, and one peptide per protein was used for quantitation with the others acting as confirmation of the protein presence (Table 1).

**Figure 2:**
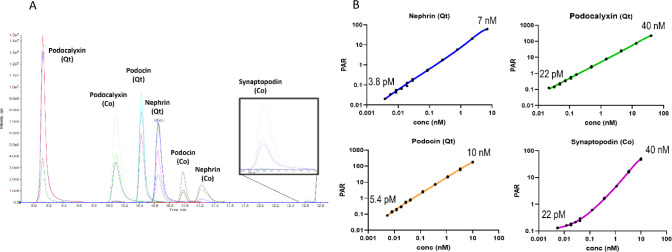
Multiplexed quantitation of podocyte peptides in urine was demonstrated with picomolar sensitivity. A) Representative extracted ion chromatogram from peptide IA-LC-MS/MS of quantitative (Qt) and confirmatory (Co) peptides. B) Calibration curves plotted as concentration (nM) versus peak area ratio (PAR) from the quantitative peptides for nephrin, podocin, podocalyxin, and synaptopodin. The upper and lower limit of quantitation (ULOQ and LLOQ) are shown at the top and bottom of each plot.

A representative extracted ion chromatogram showing the quantitative and confirmatory peptides is presented in Figure 2a, and chromatograms of STDs, QCs and blanks are also shown in Figure S2.

The peptide IA-LC-MS/MS assay performance was determined by analyzing three QC samples in six replicates on three separate days and comparing the measured value against the nominal concentration. The calibration standards passed acceptance criteria with a quantitation range that extended from 3.8 pM to 7 nM for nephrin, 22–40 nM for podocalyxin, and 5.4–10 nM for podocin (Figure 2b). Synaptopodin was targeted but not observed endogenously in HVs, disease samples, or QCs and therefore no data is reported. The confirmatory peptides for nephrin, podocalyxin, and podocin were detected in all samples for which measurable concentrations were reported, which provided additional confidence in the data.

The assay carryover was evaluated by injecting blanks after high calibration standards and high spike QCs and was found to be negligible.

The intra- and interplate RE and CVs for nephrin, podocalyxin, and podocin in the QC samples were within the ±30% limit established for this assay (Table 2).

**Table 2:** Summary of Intra- and Inter-Plate Precision and Relative Error Based on the Quantitative Peptides in Quality Control (QC) Solutions A, B, and C

	Precision (%CV)		Relative error (%RE)	
**Peptide**	**intraplate**	**interplate**	**intraplate**	**interplate (QC-C)**
Nephrin-ELV	7.0 to 27.2	9.1 to 17.2	–8.2 to 10.6	7
Podocalyxin-LGD	2.1 to 14.1	10.4 to 12.1	–10.4 to 9	–3.8
Podocin-APA	5.4 to 20.7	11.7 to 20	–16.1 to 16.1	–3

**Figure 3:**
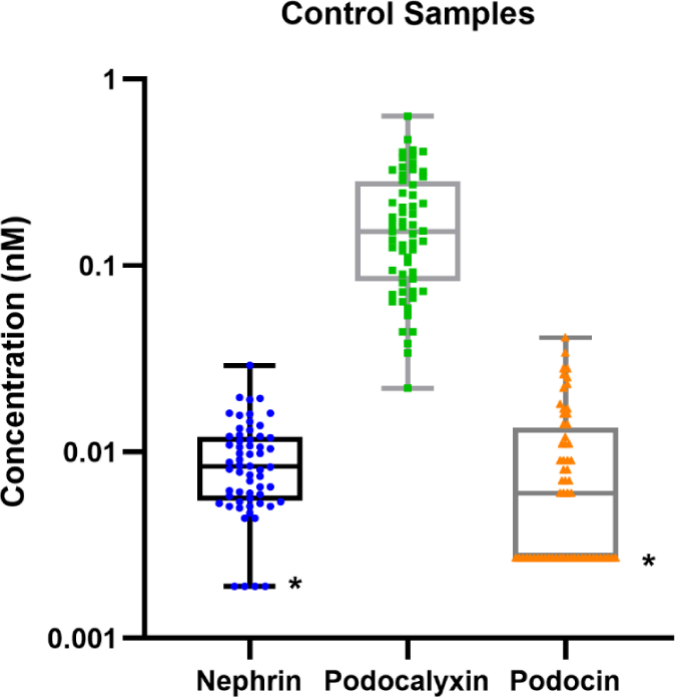
Measurements of nephrin, podocalyxin, and podocin in 60 nondisease control samples. The lower and the upper hinges of the box plot correspond to the first and third quartiles. The whiskers represent the minimum and maximum values. Samples below the lower limit of quantitation (LLOQ) were imputed as 0.5*LLOQ and are marked with (*).

The results for each run and averages are presented in Table S5 and Figure S3 designated as plate 1, 2, or 3. The RE and CV assessments were repeated three additional times (designated as plate 4, 5, or 6 in Figure 3) for a total of six plates in the span of six months showing similar performance as during qualification and plotted together to illustrate the assay performance over time.

The assay recovery in kidney disease urine was evaluated using a high spike of a known concentration of the target synthetic peptide in two separate preparations consisting of a pool of FSGS samples of a high (10 mg/mL) or low (1 mg/mL) total protein concentration. Recovery ranged from 99% to 121% for nephrin, 106% to 114% for podocalyxin, and 98% to 123% for podocin (Table S6).

Sample stability was evaluated for 3 days in separate freeze/thaw cycles, 2 and 4 h at RT or 4 °C, and on the LC autosampler at 10 °C for up to 96 h. The observed CV and RE for any analyte after 3 freeze/thaw cycles were no larger than 23% and 23%, respectively. The CV and RE at 4 °C were 22% and 23%. The maximum CV and RE after 2 h at RT were 13% and 21% and increased to 19% and −31% after 4 h. Post processing, the digested peptides had a maximum CV and RE of 26% and 28% after 96h in a 10 °C autosampler (Table S7).

### Quantitation of Podocyte Biomarkers in Urine

The qualified peptide IA-LC-MS/MS method was used to quantify nephrin, podocalyxin, and podocin in 60 urine samples from HVs. The mean concentrations for nephrin, podocalyxin, and podocin were found to be 9.34 (SD = 5.1), 188.06 (SD = 130.4), and 9.28 (SD = 9.1) pM, respectively. The median, LLOQ, Q1, Q3, and IQR are presented in Table S8. The lower limit of quantitation (LLOQ) for each peptide was defined as the lowest point on the calibration curve with a coefficient of variation <30% across two technical replicates by calculating the bias of the measured value relative to the expected value at each dilution (parallelism).^21^ No values were extrapolated below the LLOQ that also operates as the lower limit of detection (LLOD). For all the results below the limit of quantitation, the target concentrations were imputed as 0.5*LLOQ for the summary statistics and graphical presentation. There was no statistical difference in nephrin, podocalyxin, and podocin concentrations between sex or age groups (Figure S4). As before, synaptopodin was not detected. All 60 HV urine samples yielded a quantifiable podocalyxin concentration, while 93% of the nephrin measurements (56/60) and 53% of the podocin measurements (32/60) were within the quantifiable range. A side-by-side comparison between the measurements from the three analytes (Figure 3 and Table S9) showed that nephrin and podocin exhibited a similar concentration range, while podocalyxin on average was approximately 10–20 fold higher. The Pearson correlation among nephrin, podocalyxin, and podocin is presented in Figure S5. Nephrin and podocalyxin exhibiting the highest correlation (*r* = 0.6329, *p* < 0.0001) followed by podocalyxin and podocin (*r* = 0.5513 *p* = 0.0011), and nephrin and podocin (*r* = 0.3638 *p* = 0.0407).

Eighteen FSGS and two MCD samples were also analyzed using the qualified assay, and results were comparable to HV urine samples. For nephrin, podocalyxin, and podocin, the mean concentrations were 12.86 (SD = 20.8), 145.90 (SD = 216.5), and 29.76 (SD = 61.54) pM, respectively. The median, LLOQ, Q1, Q3, and IQR are presented in Table S8. Similarly, podocin was detected in 65% of samples (13/20), while nephrin and podocalyxin were quantifiable in 100% and 95% of samples (20/20 and 19/20, respectively), as shown in Table S10. The values below the LLOQ were also imputed as 0.5*LLOQ.

**Figure 4:**
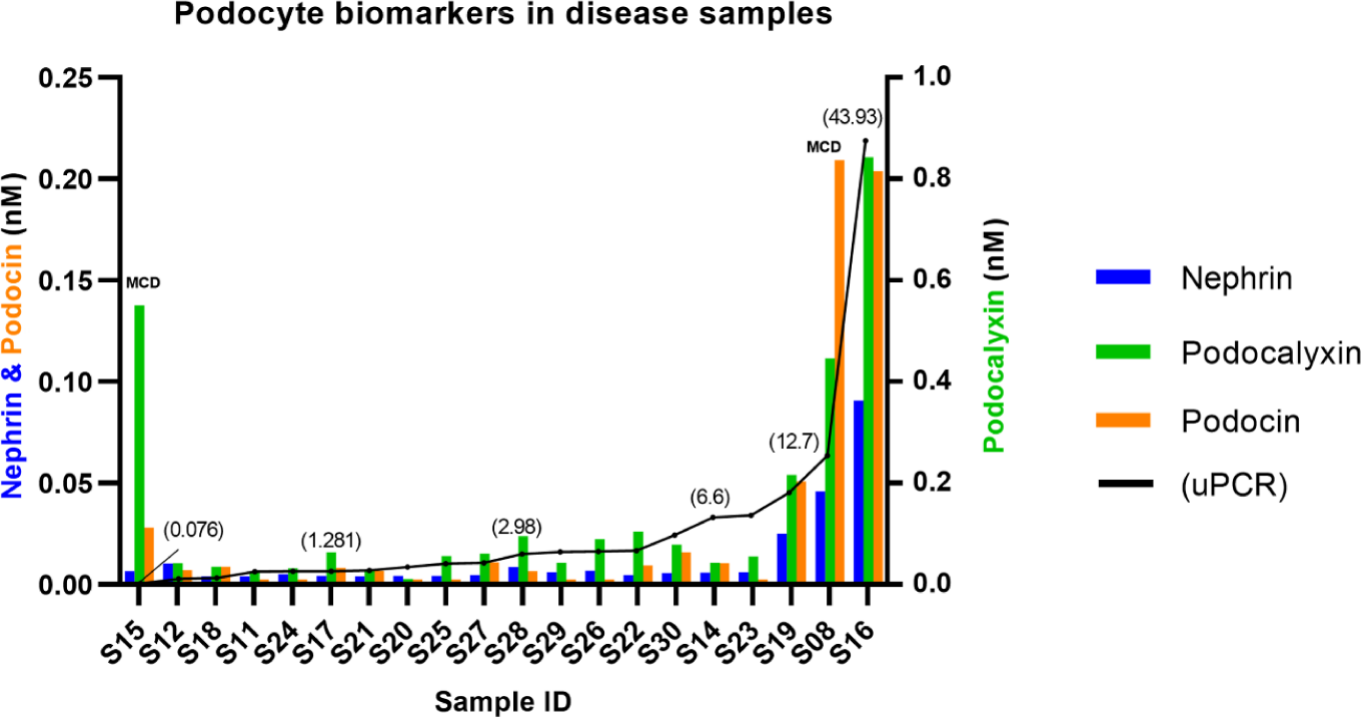
Podocyte peptides in 18 Focal Segmental Glomerulosclerosis (FSGS) and 2 Minimal Change Disease (MCD) human urine samples. Absolute concentration (nM) of nephrin and podocin is displayed in the left *y*-axis, and podocalyxin in the right *y*-axis. Samples are organized from left to right in increasing urine protein to creatinine ratio (uPCR) values, ranging from 0.076 to 43.93 (black line).

The concentrations of nephrin, podocalyxin, and podocin in the FSGS and MCD samples were organized by increasing the uPCR score (Figure 4). The trends for the three proteins recapitulated the overall uPCR score except for the sample with the lowest uPCR (S15) corresponding to one of the two MCD samples in the set, which displayed a high podocalyxin concentration. The second MCD sample, S8, also revealed a high concentration of the three proteins and a corresponding high uPCR score.

Correlation analysis (Figure S6) shows that nephrin and podocin exhibit the highest correlation (*r*= 0.9175, *p* < 0.0001), followed by nephrin and podocalyxin (*r*= 0.8561 *p* < 0.0001), and podocalyxin and podocin (*r* = 0.8139 *p* = 0.0007).

## Discussion

Monitoring glomerular protein biomarkers in urine is an attractive, noninvasive approach that could provide orthogonal and potentially earlier information to evaluate glomerular health in CKD patients and complement uPCR or serum Cr and CysC levels that increase as CKD progresses. To address this, we chose to develop an assay to simultaneously measure levels of three glomerular proteins with peptide IA-LC-MS/MS, each localized in distinctive areas of the podocyte structure: the slit diaphragm (nephrin), the podocyte surface (podocalyxin), and the cytosol interface (podocin). The target proteins excreted in urine might not be intact or properly folded in the complex urine matrix, and therefore, the use of antibodies directed against the proteins was not considered as the analytical approach. The combined use of peptide immunoaffinity, liquid chromatography, and mass spectrometry achieved high selectivity, specificity, and sensitivity within a single technique. Peptide antibody enrichment followed by reverse phase peptide separation removed analytical interferences from high abundant urinary proteins to focus on specific glomerular peptides and corresponding SIL peptide analogs.

Leveraging these capabilities resulted in a multiplex methodology that reliably quantified the three podocyte analytes down to picomolar concentrations. While podocalyxin is a surface protein present at high picomolar concentrations in urine, a high level of analytical sensitivity was necessary for the detection of podocin and nephrin. We detected the three analytes in levels comparable to those in the existing literature. For example, the relative concentrations of podocyte proteins in urine reported by Michon et al.^13^ were comparable to our findings in HVs and disease samples with podocalyxin as the highest followed by nephrin and podocin. Biarc et al.^15^ found podocalyxin ranged from 15.2 to 44.2 ng/mL in eight HVs, comparable to the 1.30–37.02 ng/mL (units converted using podocalyxin MW) we identified in 60 samples from HVs. Likewise, Martineau et al.^14^ found podocin in preeclampsia samples ranging from undetectable to 8.1 pmol/mmol creatinine units and in Fabry disease samples from undetectable to 23 pmol/mmol creatinine units. Our measurements in HV samples are in general agreement, with positive detection in 32/60 samples ranging from 0.09 to 3.75 pmol podocin/mmol creatinine). Nephrin was targeted by van Duijl et al.^16^ using LC-MS/MS with a calibration range of 5.4 to 1034 pM but was not detected in urine from ten HVs and 20 disease samples. Our method monitors a different peptide sequence, includes peptide-based immunoaffinity, and uses a higher urine volume that enabled the measurement of nephrin in HVs (1.9 to 29.1 pM) and disease samples (4.02 to 90.8 pM) (Tables S9 and S10). These nephrin measurements were supported by a good correlation with podocalyxin and podocin, which shows consistency between the three independent podocyte analytes (Figures S5 and S6).

The method presented here utilizes 100 μL of clarified urine, similar to what is typically used for Cr and CysC measurements. The CV and RE, 3-day freeze thaw, and pre- and post-sample processing stability were evaluated for each analyte and found suitable for the analysis of samples in 96-well plate format with good reproducibility. The methodology was also suitable for the analysis of healthy and disease samples with a wide range of uPCR measurements (0.076 to 43.93) showing a good analyte recovery and detectability even in the presence of high concentrations of urinary proteins. The measurements of three podocyte-related proteins with different locations and functions exhibited a good correlation between them. This correlation was stronger in disease samples where more protein shedding is expected, which suggests that podocyte biomarkers could provide additional insights into the health of the filtration barrier by measuring the presence of podocyte excretions in urine (podocyturia).

The detection of glomerular proteins in urine is of high interest as an approach to understanding the biology of glomerular kidney disease and aiding the development of biomarkers that can be used to diagnose and stratify patients. This method could be applied to detect the excretion of podocyte proteins in relevant CKD subgroups, longitudinal samples, or controls to identify their levels in different types and stages of glomerular damage. This would provide a better understanding of glomerular injury and CKD onset and progression and potentially move toward early detection and intervention or aid in the testing of novel therapeutic agents or patient stratification. Measurements from larger or longitudinal sample sets across a range of kidney diseases with distinct pathologies and disease severity will further establish the translational utility of the assay.

## Data Availability

The targeted LC-MS/MS data generated in this study has been deposited in the MassIVE data repository under massive.ucsd.edu with project identifier MSV000094428.

## Supporting Information Available

Supplemental methods: Chemicals and peptides. Antipeptide antibody generation and column preparation. Urine clarification, protein precipitation and total protein quantitation. Pellet predigestion. SIL addition and protein digestion. Peptide Immunoaffinity LC-MS/MS. Calibration curves and QC samples. Data processing. Figure S1. Peptide immunoaffinity LC configuration. Figure S2. Sample Chromatograms of QCs, calibration standards. Figure S3. Relative accuracy and precision evaluation. Figure S4. Quantification of nephrin, podocalyxin and podocin in 60 urine samples from HV. Figure S5. Pearson correlation coefficient in 60 nondisease control urine samples. Supplemental Figure 6. Pearson correlation coefficient in 20 disease samples (PDF)
Table S1. Chromatography gradient. Table S2. MS method. Table S3. Calibration curve range for each study analyte. Table S4. Quality control samples (QC). Table S5. Three-day validation results. Table S6. Analyte recovery in FSGS matrix samples Table S7. Sample stability. Table S8. Sample statistics, control (a) and disease (b) in pM concentrations Table S9. Absolute quantitation of nephrin, podocin and podocalyxin in 60 nondisease urine control samples. Table S10. Absolute quantitation of nephrin, podocin and podocalyxin in 18 FSGS and 2 MCD samples (XLSX)

## Abbreviations

CKD: Chronic kidney disease
GFR: glomerular filtration rate
Cr: Creatinine
CysC: Cystatin C
uPCR: urine protein to creatinine ratio
LC-MS/MS: Liquid chromatography tandem mass spectrometry
LLOQ: lower limit of quantitation
HV: Healthy volunteer
SIL: Stable isotope labeled
FSGS: Focal segmental glomerulosclerosis
MCD: Minimal change disease
STD: Calibration standards
QC: Quality control
CV: Coefficient of variation (precision)
RE: Relative error (relative accuracy)
IA: Immunoaffinity
TS: Transition summing
TEA: Triethanolamine
ELISA: Enzyme-linked immunoassay
TFA: Trifluoroacetic acid
Ambic: 100 mM ammonium bicarbonate pH = 8
MRM: Multiple reaction monitoring
PAR: Peak area ratio
