# Supplementary material for: Development of a Multiplexed LC-MS/MS Assay for the Quantitation of Podocyte Injury Biomarkers Nephrin, Podocalyxin, and Podocin in Human Urine
Source: J Proteome Res. 2024 Dec 9;24(1):282–8. doi: 10.1021/acs.jproteome.4c00751 (PMC11705212; doi:10.1021/acs.jproteome.4c00751)
Supplement: Supplementary file 1 — pr4c00751_si_001.pdf [file pr4c00751_si_001.pdf]

Supplemental Material

TITLE: Development of a multiplexed LC-MS/MS assay for the quantitation of podocyte injury biomarkers nephrin, podocalyxin, and podocin in human urine.

AUTHORS: Carlos A Morales-Betanzos[1], Stephen P Berasi[2], Joel D Federspiel[1], Hendrik Neubert[1], Mireia Fernandez Ocana*[1].

Affiliations list:

1 Pfizer Inc., Andover, Massachusetts 01810, United States.

2 Pfizer Inc., Cambridge, MA 02139, USA.

***Corresponding author:**

Dr. Mireia Fernandez Ocana, G1008B, 1 Burtt Road, Pfizer Inc., Andover, Massachusetts 01810, United States. +1 (978) 2472844. Mireia.FernandezOcana@pfizer.com

Table of Contents:

Supplemental methods

Chemicals and peptides

Anti-peptide antibody generation and column preparation.

Urine clarification, protein precipitation and total protein quantitation.

Pellet pre-digestion.

SIL addition and protein digestion.

Peptide Immunoaffinity LC-MS/MS.

Calibration curves and QC samples.

Data processing.

Supplemental Tables

Supplemental Table 1. Chromatography gradient.

Supplemental Table 2. MS method.

Supplemental Table 3. Calibration curve range for each study analyte.

Supplemental Table 4. Quality control samples (QC).

Supplemental Table 5. Three-day validation results.

Supplemental Table 6. Analyte recovery in FSGS matrix samples

Supplemental Table 7. Sample stability.

Supplemental Table 8. Sample statistics, control (a) and disease (b) in pM concentrations

Supplemental Table 9. Absolute quantitation of nephrin, podocin and podocalyxin in 60 non-disease urine control samples.

Supplemental Table 10. Absolute quantitation of nephrin, podocin and podocalyxin in 18 FSGS and 2 MCD samples.

Supplemental Figures:

Supplemental Figure 1. Peptide immunoaffinity LC configuration.

Supplemental Figure 2. Sample Chromatograms of QCs, calibration standards.

Supplemental Figure 3. Relative accuracy and precision evaluation.

Supplemental Figure 4. Quantification of nephrin, podocalyxin and podocin in 60 urine samples from HV.

Supplemental Figure 5. Pearson correlation coefficient in sixty non-disease control urine samples.

Supplemental Figure 6. Pearson correlation coefficient in twenty disease samples.

Supplemental References.

Supplemental methods.

Chemicals and peptides.

Acetonitrile (AC615140025), ammonium bicarbonate (A643-500), formic acid (A117-50), sodium azide 10% (S0209), TPCK-trypsin (20233), and isopropanol (A451-4) were acquired from Fisher. Acetone (270725), ammonium formate (78314), dimethyl pimelimidate (DMP, D8388), trifluoroacetic acid (A116), rat serum (R9759), triethanolamine (TEA, T1377), Trizma Pre-Set Crystals, pH 8.3 (T8943), ethanolamine (411000), and urea (51457) were acquired from Sigma Aldrich. BCA protein kit (23227), single-use dithiothreitol (DTT, A39255) and iodoacetamide (IAM, A39272) were acquired from Thermo Scientific. Unlabeled 'light' peptide and SIL peptide standards were acquired from New England Peptide and Thermo Scientific (>95% purity; concentration determined by amino acid analysis).

Anti-peptide antibody generation and column preparation.

Affinity purified rabbit polyclonal antibody specific for the targeted peptide sequences were acquired from Cambridge Research Biochemicals, as previously described [1, 2]. In summary, New Zealand white rabbits were injected with peptides conjugated to keyhole limpet hemocyanin (KLH) in the presence of adjuvant. The sera from the immunized animals were collected and screened for reactivity using ELISA and pooled based on the antibody titers. The pooled antiserum was peptide ligand affinity purified and the antibody concentration quantified. As per vendor specifications, all procedures were performed in accordance with Institutional Animal Care and Use Committee regulations.

The preparation of anti-peptide antibody columns has been previously described [3-6]. Briefly, antibodies were immobilized in Protein G agarose beads using 270 µL of Poros 20 Protein G slurry (Applied Biosystems cat #1-5128-10) packed into a 2.1 mm cartridge (IDEX Health & Science Upchurch cat # 5050IP0502100320) using a stainless-steel UHP column packing system (Teledyne SSI Cat#150054). The column was washed with water and 100 mM sodium acetate, pH 5.5. A mix of 0.2-0.7 mg of three to four antibodies adding up to approximately 1.8 mg of total antibody per column was recirculated overnight in 3-5 mL of 100 mM sodium acetate in a closed loop system. The following day the column was washed with PBS, and the antibodies cross-linked to the agarose beads with 7.78 mg/mL dimethyl pimelimidate (DMP) in 100 mM triethanolamine (TEA). The excess crosslinker was removed with 100 mM ethanolamine pH 8.0, and the column was washed with 200 mM Tris, pH 8.3 to block any remaining crosslinker. The column was then washed with 0.5% TFA followed by 25 mM ammonium formate to remove any residual crosslinker before use.

Urine clarification, protein precipitation and total protein quantitation.

Urine samples were clarified by centrifugation at 1,000 × g for 5 minutes at 4 ºC, and the supernatant was transferred to low protein binding Eppendorf tubes in 100 µL aliquots. The clarified urine samples were stored at -80 °C until needed. 100 µL of clarified urine was incubated with 400 µL of cold acetone (4 °C) for 90 minutes on ice. Proteins were precipitated by centrifugation at 15,000 × g for 45 minutes at 10 ºC. The supernatant was removed, and the pellets dried for 20 minutes at room temperature (RT). One protein pellet per sample was resuspended in 500 µL PBS and shaken for 10 minutes. The

protein concentration was determined using a BCA assay following the manufacturer's instructions.

Pellet pre-digestion.

Clarified urine samples with high protein concentrations were diluted to below 4 mg/mL with PBS, while clarified urine samples with lower protein concentration were used undiluted. A separate aliquot of 100 µL of clarified undiluted or diluted urine was precipitated as described above, and 100 µL of 2.5 µg/µL of trypsin in 100 mM ammonium bicarbonate pH 8 (Ambic) was added to all pellets. Samples were incubated at 37 °C with shaking overnight.

SIL addition and protein digestion.

SIL peptides were added to the following final concentrations in the sample; nephrin: 0.1 nM ELV, 0.2 nM DGL; podocin: 0.07 nM APA, 0.5 nM TQG; podocalyxin: 0.3 nM LGD, 1 nM DDL; synaptopodin 0.6 nM VTP. Reduction and alkylation were performed with DTT at 60 ºC at a concentration of 5.5 mM and IAM at RT at a concentration of 11 mM, respectively, followed by the addition of 8 M urea to a final concentration of 1 M. The samples were digested by adding 150 µL of trypsin to a final concentration of 0.33 µg/µL and incubated with shaking overnight at 37°C.

Peptide Immunoaffinity LC-MS/MS.

The peptide immunoaffinity strategy previously described was used [2, 3, 5]. A diagram of the LC configuration is presented in Supplemental Figure 1. Briefly, 100 µL of digested samples were loaded into the antibody columns in 25 mM ammonium formate, and unbound peptides and contaminants were removed to waste with a 300 mM

ammonium formate wash. Bound peptides were eluted with 0.5% TFA into a C18 pre-column (Thermo Scientific Cat# 160454). Chromatography separation was accomplished using a 5 to 30% acetonitrile 7-minute gradient at a flow rate of 0.6 µL/min (Supplemental Table 1) on an Acclaim PepMap 15 cm, nanoviper column (Thermo Scientific Cat# 164534) at 70 ºC.

The eluate from the nanoflow chromatography was introduced into a Sciex 6500+ triple quadrupole mass spectrometer (AB Sciex, Framingham MA, USA) operating in positive ion mode with an interface heater temperature of 180 ºC, a spray voltage of 3200-3600 V, and an ion gas source of 5-12. Detection was accomplished using a scheduled multiple reaction monitoring (MRM) method with cycle and dwell time selected to achieve a minimum of 15 points per peak. The transition list, typical retention times, cycle and dwell time information are provided in Supplemental Table 2. In order to further enhance the signal-to-noise ratio and corresponding sensitivity of nephrin ELV, we made use of transition summing (TS) [7, 8]. In this approach, the top ELV precursor ion to fragment transition is scanned in five independent acquisitions during each MRM cycle, and the five spectra are summed together to generate a single, quantifiable area under the curve.

Calibration curves and QC samples.

QC samples consisted of three pools of urine designated QC-A, -B, and -C with discrete concentrations of the target peptides. QC-A is a pool of longitudinal urine samples from a single HV. QC-B is a mixture of urine from HVs and diabetic nephropathy subjects. QC-C was made from QC-B spiked with the target peptides at a concentration of 0.066 nM for nephrin and 1 nM for podocalyxin and podocin (Supplemental Table 4).

Dilution of urine samples with high protein concentration

Application of a preliminary version of this assay to kidney disease (MCD and FSGS) samples showed only low-quality LC-MS/MS signals compared to HV control urine. It was determined that the high protein content in the kidney disease samples saturated trypsin digestion and interfered with the antibody columns during the online peptide enrichment. During an initial analysis, samples with a high protein content i.e., samples 8, 16, and 19, (Supplemental Table 10) exhibited no signals from either endogenous protein or internal standards. These challenges were overcome by diluting the samples containing high protein levels with PBS to a maximum final concentration of 4 mg/mL before the acetone precipitation step. This concentration was determined as the maximum concentration usable with the selected digestion conditions and compatible with the immunoaffinity column to allow for quantifiable signals for the internal standard and endogenous peptides. All dilution factors were recorded, and the initial analyte concentration was back-calculated. In addition, a pre-digestion step with trypsin overnight was incorporated into the workflow to increase the digestion efficiency of the protein pellets.

Data processing.

The peak area integration was conducted using MultiQuant 3.0 or higher (Sciex, Framingham MA). Integration parameters were established using the peptide standards and applied consistently across all samples: Gaussian smooth width 0.7, noise percentage 70%, and peak split of 2 points. The peak areas of the light peptides were divided by the area of the SIL peptides to obtain the peak area ratio (PAR). The PAR from all targets were exported into GraphPad Prism 9.5 or higher (Dotmatics, Boston MA) for

statistical analysis. The calibration curves were fit using a sigmoidal model with a weighted sum of the squares of $1/X^2$ in GraphPad, and the interpolated concentration values were calculated. The results were assembled in Microsoft Excel, and the precision (percent coefficient of variation, %CV) and relative accuracy (percent relative error, %RE) were calculated for all STDs and QC samples.

Supplemental Figures.

Supplemental Figure 1. Peptide immunoaffinity LC configuration.

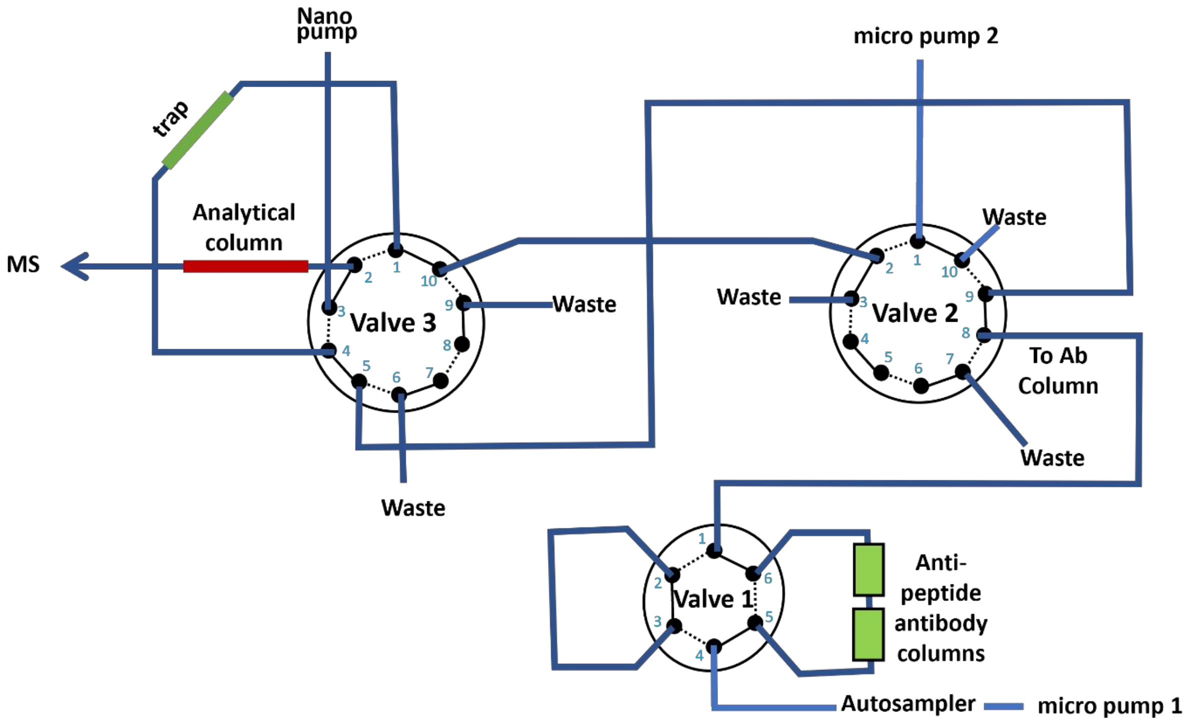

Supplemental Figure 2. Selected sample chromatograms of blanks, quality controls solutions (QCs), calibration standards, and healthy volunteers.

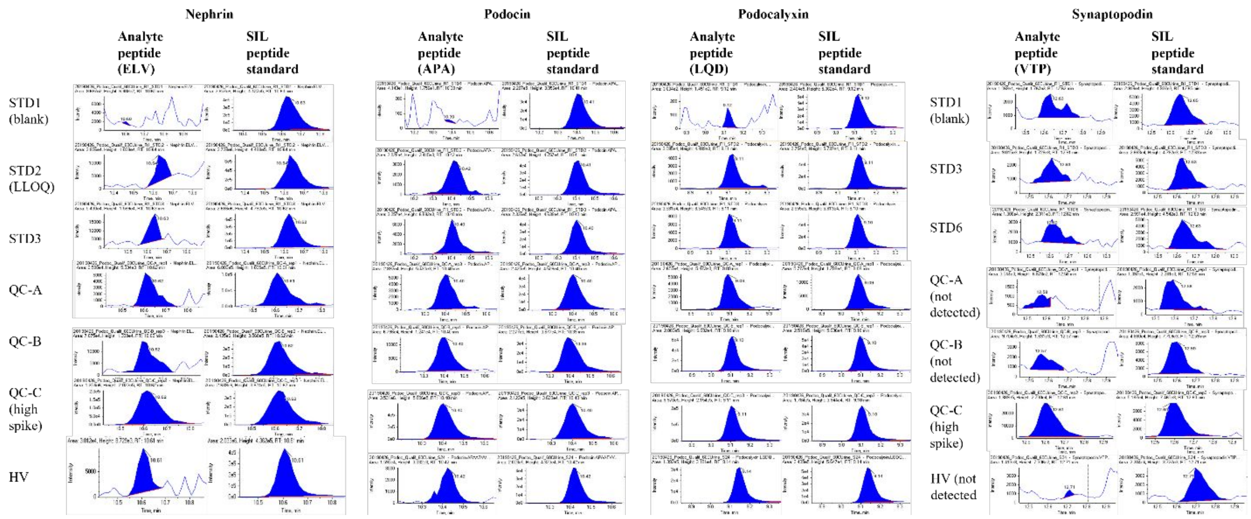

Supplemental Figure 3. Relative accuracy and precision evaluation. Measurements of three QC samples were conducted in six replicates in six independent plates. Plots 1, 2, and 3 are qualification plates. Plots 4, 5, and 6 are QC samples from subsequent uses of the assay after qualification. (a) %RE were calculated and plotted together for each run across all QC types. Acceptance criteria was set at ±30% (dotted bar). Median is presented as a solid bar. (b) Precision was calculated for each QC type in six replicates and plotted per batch run.

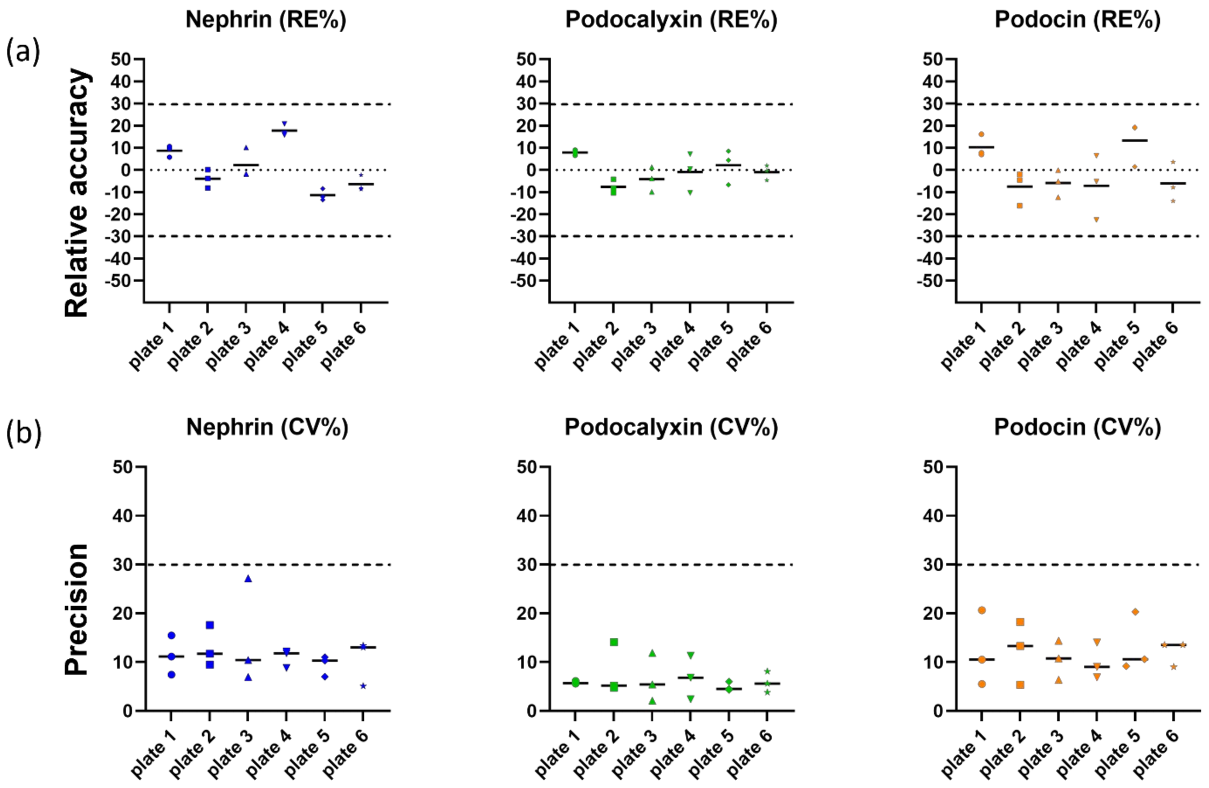

Supplemental Figure 4. Quantification of nephrin, podocalyxin and podocin in 60 urine samples from HV plotted by age or sex showed no significant difference between the groups. Samples below LLOQ were imputed as 0.5*LLOQ and are marked with (*)

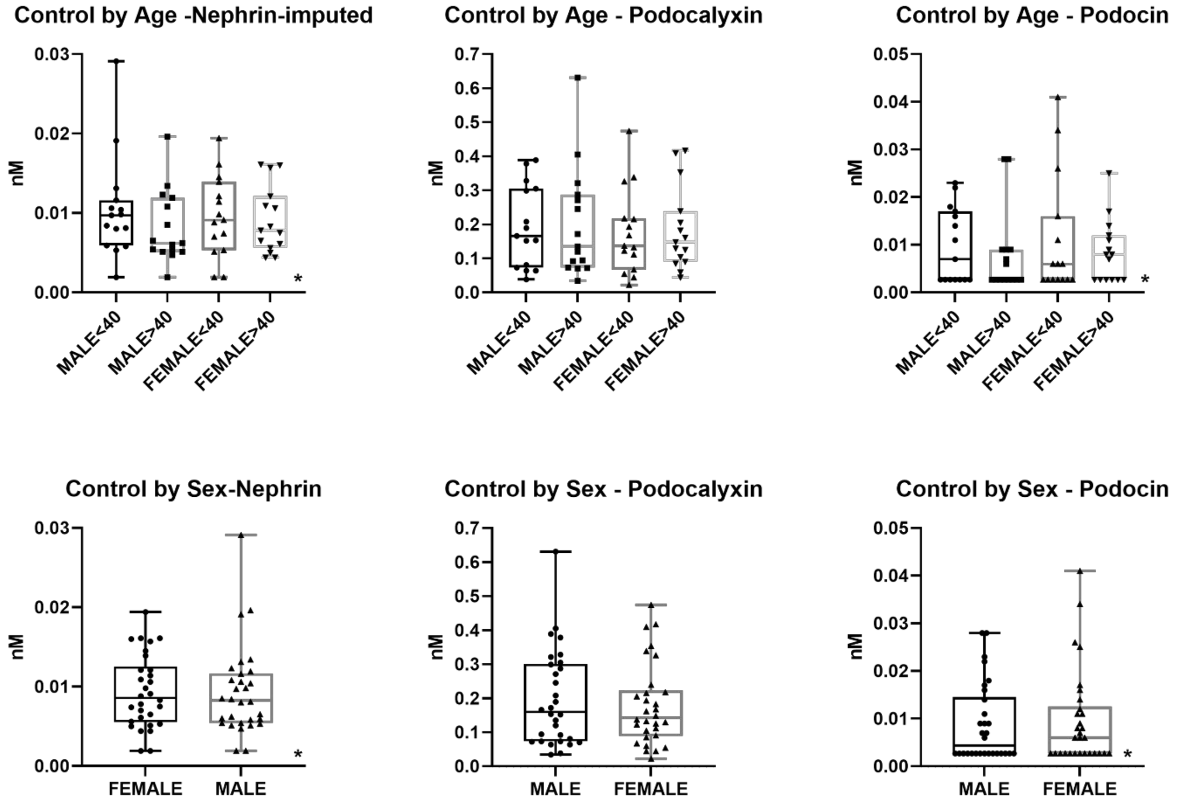

Supplemental Figure 5. Pearson correlation coefficient with a 95% confidence interval significance was calculated between the three podocyte biomarkers in sixty non-disease control urine samples.

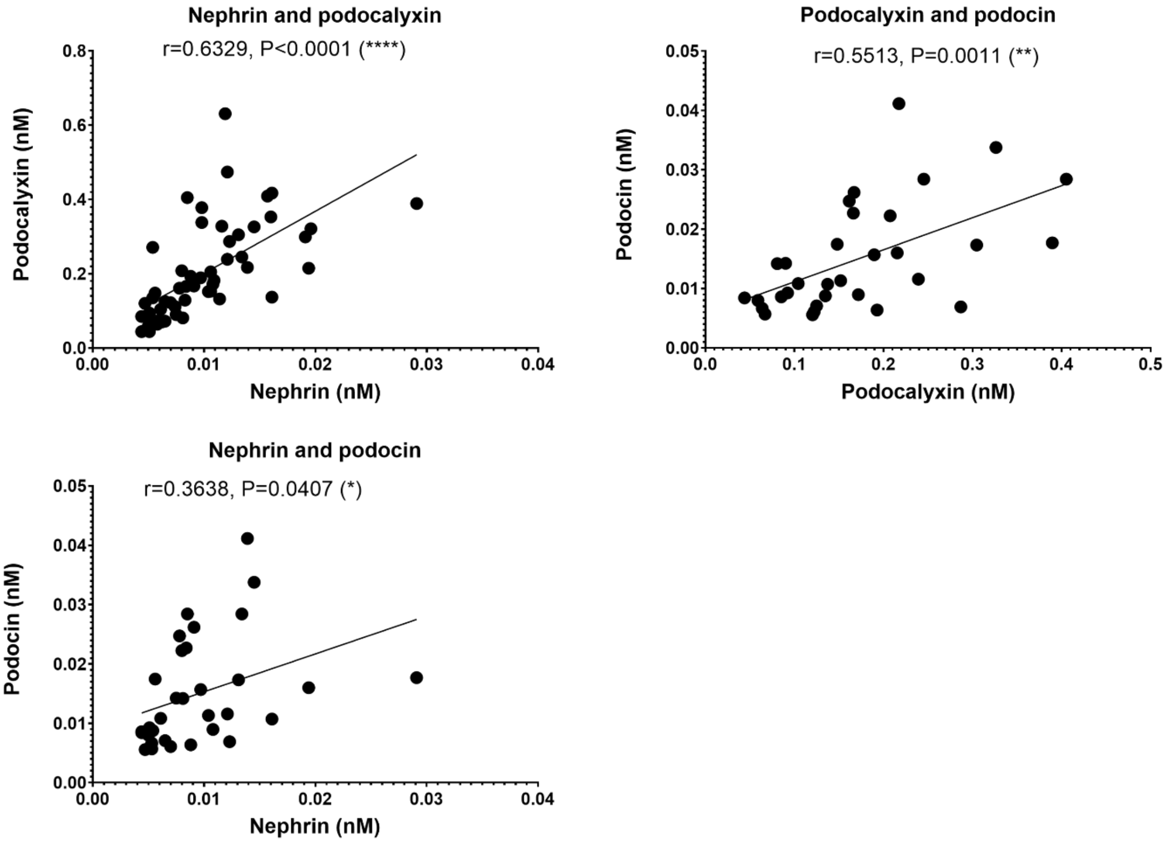

Supplemental Figure 6. Pearson correlation coefficient with a 95% confidence interval significance was calculated between the three podocyte biomarkers in twenty disease samples.

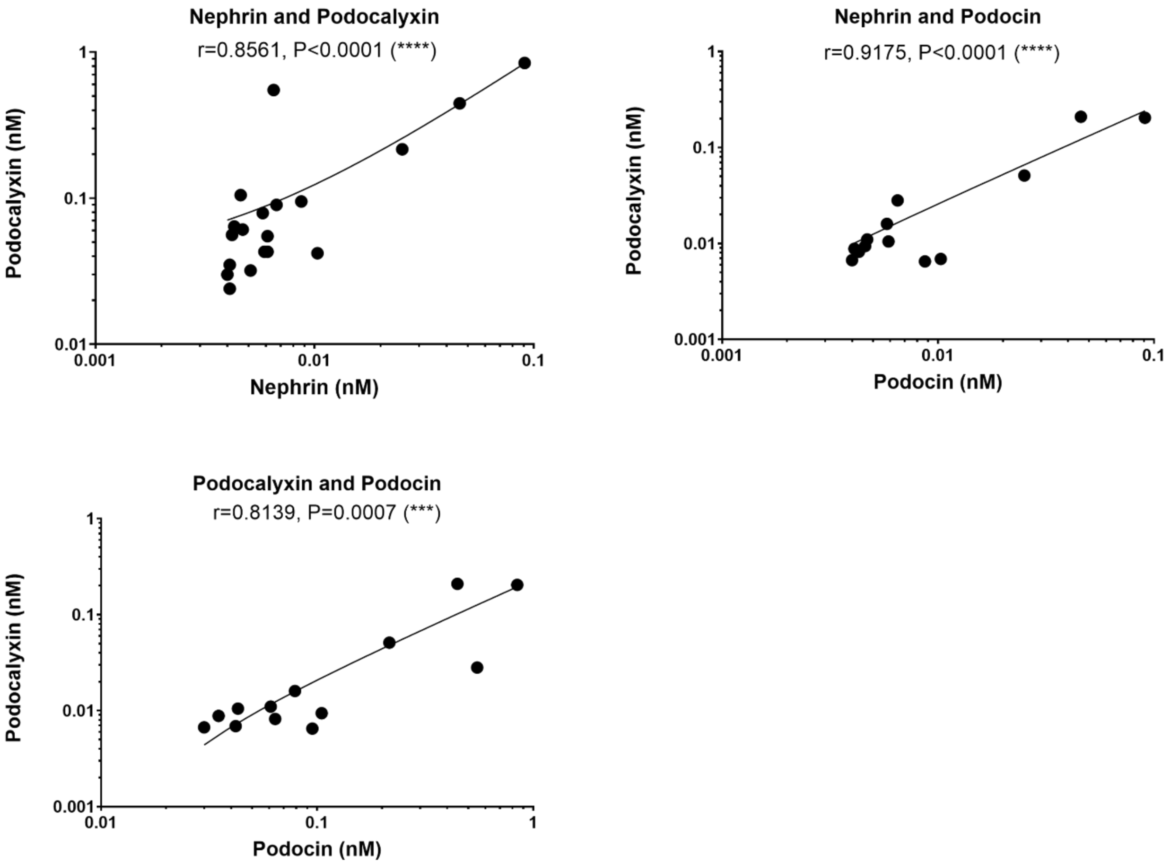

Supplemental references.

1. Fan, Y. Y.; Farrokhi, V.; Caiazzo, T.; Wang, M.; O'Hara, D. M.; Neubert, H., Human FcRn Tissue Expression Profile and Half-Life in PBMCs. *Biomolecules* **2019,** *9* (8).
2. Dufield, D. R.; Radabaugh, M. R., Online immunoaffinity LC/MS/MS. A general method to increase sensitivity and specificity: How do you do it and what do you need? *Methods* **2012,** *56* (2), 236-45.
3. Neubert, H.; Gale, J.; Muirhead, D., Online high-flow peptide immunoaffinity enrichment and nanoflow LC-MS/MS: assay development for total salivary pepsin/pepsinogen. *Clin Chem* **2010,** *56* (9), 1413-23.
4. Neubert, H.; Muirhead, D.; Kabir, M.; Grace, C.; Cleton, A.; Arends, R., Sequential protein and peptide immunoaffinity capture for mass spectrometry-based quantification of total human beta-nerve growth factor. *Anal Chem* **2013,** *85* (3), 1719-26.
5. Neubert, H.; Shuford, C. M.; Olah, T. V.; Garofolo, F.; Schultz, G. A.; Jones, B. R.; Amaravadi, L.; Laterza, O. F.; Xu, K.; Ackermann, B. L., Protein Biomarker Quantification by Immunoaffinity Liquid Chromatography-Tandem Mass Spectrometry: Current State and Future Vision. *Clin Chem* **2020,** *66* (2), 282-301.
6. Palandra, J.; Finelli, A.; Zhu, M.; Masferrer, J.; Neubert, H., Highly specific and sensitive measurements of human and monkey interleukin 21 using sequential protein and tryptic peptide immunoaffinity LC-MS/MS. *Anal Chem* **2013,** *85* (11), 5522-9.
7. Pauwels, S.; Jans, I.; Peersman, N.; Billen, J.; Vanderschueren, D.; Desmet, K.; Vermeersch, P., Possibilities and limitations of signal summing for an immunosuppressant LC-MS/MS method. *Anal Bioanal Chem* **2015,** *407* (20), 6191-9.
8. Johnson, J. S.; Palandra, J.; Psychogios, N.; Walsh, J. M.; Neubert, H., Improving the Sensitivity of Protein Quantification by Immunoaffinity Liquid Chromatography horizontal line Triple Quadrupole Mass Spectrometry Using an Iterative Transition Summing Technique. *Anal Chem* **2024**.
